# Further Experience with Balsamo Del Deserto

**Published:** 1895-10

**Authors:** W. H. White

**Affiliations:** Silver City, N. M.


### FURTHER EXPERIENCE WITH BALSAMO DEL DESERTO.

BY DR. W. H. WHITE, SILVER CITY, N. M.

After another year of added experience in the use of balsamo del deserto in dental operations, and at the risk of being judged an enthusiast on the subject, I will say that I had now rather do without any other article of the Dental Materia Medica than this. The record is still unbroken: I have never heard of a single case of recurrence of apical irritation after I have filled the roots with this material.

I believe the real secret of success with this material is,—

1. That it is soft and permanently remains soft, and that it will adhere to a damp surface. A root-canal is like a tube, and is liable to change of calibre from thermal or other causes. When filled with a hard substance and such changes occur there must form a crack between the filling and canal-wall, or within the substance of the filling, thus allowing septic invasion; but when such a canal is filled with balsamo, the substance of the filling gives without cracking when there is a change of calibre, and when you add its permanent antiseptic and local antiphlogistic qualities and its entire compatibility with tissue of all kinds there seems wanting nothing to be desired for these purposes.

I have discarded the use of the barbed nerve-extractor in cases of recently-destroyed pulps. I take out the bulbous portion with engine-burs, and clean the bulbous half of the root-canal with the Brewer drill, purposely leaving the dead nerve undisturbed in the apical half. After three years' experience I have not known a single case to cause trouble; the absolute immunity from all pain, soreness, neuralgia, and abscess is certainly remarkable in the light of my former experience in such cases.

My usual practice is to fill roots and crowns permanently at the second sitting, or, where I use local anæsthetics, taking out the pulp, I fill permanently, root and crown, at first sitting.

I have a record of one hundred and thirty-eight abscesses cured during the past year. With these cases I have used nothing as a germicide except oil of cinnamon, and have filled all roots with the balsamo. In forty-eight of these cases the roots and, when amalgam was used, the crowns also were permanently filled within thirty hours after treatment began. In seventy-one of these cases the roots were filled on the second or third day after treatment. Many of these latter might have been filled safely within twenty-four hours, but I either did not have time to attend to them or there was no necessity for haste. The only cases found that I did not feel safe in filling within forty-eight hours were those where the canal was so small that I had great difficulty in getting the germicide into the apical space, and those where the apex was necrosed.

I found three cases of abscess during the year that I could not cure,—they all occurred in the same month: they had fistulæ with apex necrosed and a copious flow of thin, milk-white pus. I failed to cure a single abscess in this mouth by any method of treatment, even after amputating the necrosed apex.

In none of the one hundred and thirty-eight cases treated and cured did I inject any medicament into the apical space, but allowed the cinnamon to reach that space by absorption. I made no attempt to remove the dead pulp from the apical third of the root-canal. I dried the canal only so much as could be done with absorbent cotton. I used no escharotic to break up the pus-sac. I found blind abscesses far more amenable to treatment than fistulous abscesses. It was immaterial to me whether the pus was discharged or not. Of course, where the pus discharged through the canal, I would allow all to escape that way that would, and when the abscess had made an opening through the process, I would lance the gum, but made no further attempt to get rid of the pus.

The theory is that it is not the pus-cells that hinder the healing process, nor is it the dead bodies of the microbes, but it is the ptomaines, the excretions of the microbes, that cause the trouble. Therefore, if you use a germicide it kills the microbes and stops the excretion of ptomaines; and let that germicide be one that does not poison human cells, but leaves them in healthy condition, so that they may perform their proper function; then the giant-cells, the scavengers of the body, will quickly devour the pus-cells and the dead bodies of the microbes, and the leucocytes, the builders of the body, will quickly repair the breach.

Oil of cinnamon is too strong a drug to use full strength in the apical space. Leaving part of the dead nerve in the canal, so far from being a detriment, is an absolute benefit, as it allows only minute quantities of the drug to seep into the apical space.

Dentists have been taught so long that it is necessary to evacuate the pus and to break up the pus-sac with escharotics, necessary to clean the canal thoroughly of dead pulp, and to dry the canal, etc., that it is difficult to make them believe that all these processes are not only useless but detrimental.

I have used this material long enough to observe that the dead roots of temporary teeth filled with balsamo del deserto seem to be absorbed the same as live roots are. I believe the giant-cells will absorb a dead root as readily as a live root if there be no microbic ptomaines present to hinder them from performing their functions.

I have been particularly interested in watching the results of using balsamo with amalgam in filling teeth. In looking at the material one cannot realize its practical benefits. I have gradually grown into its use until I now use it in all amalgam fillings. Balsamo is so attenuant that a very small quantity of it will completely permeate the amalgam and perfectly insulate the several particles of metal, so that such a filling is as poor a conductor of heat, cold, and electricity as cement or gutta-percha. I now use it in all cases of nearly exposed pulp where capping of gutta-percha or of cement was formerly employed.

In recapitulating its good qualities I will say, first, it is more compatible with tooth-structure than any other filling-material yet devised. When a tooth is decayed so that the pericementum is exposed, mix the amalgam with balsamo, and place it lightly against this tissue, and it will remain perfectly comfortable. Any material which can be thus used must be compatible with tooth-structure.

2. I believe it hermetically seals a cavity, which cannot be said of any other filling-material now in use, there being a strata of balsamo next to the tooth that is soft and remains soft.

3. Fillings will require less undercutting than amalgam alone; in fact, they adhere to the walls of the cavity as firmly as cement.

4. When balsamo is mixed with amalgam it causes the filling to be as poor a conductor of heat, cold, and electricity as a cement filling, and is impermeable to the fluids of the mouth.

5. The tooth-edge does not crumble as it does with amalgam alone. I think this is due to its entire compatibility.

6. The filling does not blacken the tooth as amalgam fillings do, balsamo keeping the filling from oxidizing. While it is thought these salts have a preserving effect, still they are not necessary when balsamo is used.

7. It is especially useful in filling temporary teeth where it is necessary to insert fillings quickly, and often without thorough preparation of the cavity.

8. Patients never complain of uneasiness or pain from thermal changes when sensitive teeth are filled with this material. In short, I believe this filling combines all the good qualities of amalgam, gutta percha, and cement, and has a number of good qualities that none of these possess.

When I wish to fill over-exposed pericementum, or where the pulp is nearly exposed, I mix balsamo with part of the amalgam and press it down lightly with spunk folded tightly in the pliers, finishing the filling with pure amalgam. For ordinary cavities I cover the cavity first with balsamo and work the amalgam into this; this forms a pasty mass; when the cavity is half full I wipe off the surplus balsamo with spunk, firmly rubbing it against the walls of the cavity, then complete the filling with purer amalgam. When balsamo is mixed with amalgam it at first forms an unsightly mass, resembling blue mass in appearance, and shows dark through thin enamel, but after it has been in a tooth a few weeks or months it loses this dark color and the fillings look far better than if amalgam alone were used. Spunk moistened with alcohol used on the amalgam makes a clean, hard surface to these fillings.

The reason I think balsamo preferable to the various alcoholic or chloroform solutions of the gums now in use for lining cavities is that these solutions become hard on exposure to air and moisture. When lining a cavity, if the calibre of the cavity changes, these linings crack and thus allow the invasion of septic matter; balsamo will permanently retain its present consistency and cannot crack,—it is also more compatible with the tooth than these gum solutions.

Among other things for which I find balsamo useful is, first, to relieve pain in the alveola after tooth-extraction, especially where no firm blood clot is formed. A plug of cotton saturated with balsamo put into the socket will keep it free from pain until thrown off by granulation.

2. When an exposed pulp is painful and congested, the pain is relieved and the circulation is restored to its normal condition by an application of balsamo. The relief from pain is caused by antiphlogistic properties and not by anæsthetic properties. I have thus often relieved the pain caused by arsenical application.

Until lately I thought balsamo a vegetable product, but I have discovered it to be an animal product; an insect uses this material rear its young in the same way as bees use beeswax.

